# SS-31 Provides Neuroprotection by Reversing Mitochondrial Dysfunction after Traumatic Brain Injury

**DOI:** 10.1155/2018/4783602

**Published:** 2018-08-27

**Authors:** Yihao Zhu, Handong Wang, Jiang Fang, Wei Dai, Jiang Zhou, Xiaoliang Wang, Mengliang Zhou

**Affiliations:** ^1^Department of Neurosurgery, Jinling Hospital, Medical School of Nanjing University, Nanjing, Jiangsu 210002, China; ^2^Department of Neurosurgery, Jinling Hospital, School of Medicine, Southeast University, Nanjing, Jiangsu 210002, China; ^3^Department of Neurosurgery, Jinling Hospital, Nanjing Medical University, Nanjing, Jiangsu 210002, China

## Abstract

SS-31, a novel mitochondria-targeted peptide, has been proven to provide neuroprotection in a variety of neurological diseases. Its role as a mitochondrial reactive oxygen species (ROS) scavenger and the underlying pathophysiological mechanisms in traumatic brain injury (TBI) are still not well understood. The aim of the designed study was to investigate the potential neuroprotective effects of SS-31 and fulfill our understanding of the process of the mitochondrial change in the modified Marmarou weight-drop model of TBI. Mice were randomly divided into sham, TBI, TBI + vehicle, and TBI + SS-31 groups in this study. Peptide SS-31 (5 mg/kg) or vehicle was intraperitoneally administrated 30 min after TBI with brain samples harvested 24 h later for further analysis. SS-31 treatment significantly reversed mitochondrial dysfunction and ameliorated secondary brain injury caused by TBI. SS-31 can directly decrease the ROS content, restore the activity of superoxide dismutase (SOD), and decrease the level of malondialdehyde (MDA) and the release of cytochrome c, thus attenuating neurological deficits, brain water content, DNA damage, and neural apoptosis. Moreover, SS-31 restored the expression of SIRT1 and upregulated the nuclear translocation of PGC-1*α*, which were proved by Western blot and immunohistochemistry. Taken together, these data demonstrate that SS-31 improves the mitochondrial function and provides neuroprotection in mice after TBI potentially through enhanced mitochondrial rebiogenesis. The present study gives us an implication for further clinical research.

## 1. Introduction

Traumatic brain injury (TBI) is a worldwide health problem, which has caused lots of medical resources [1, 2]. The patients who suffer from TBI usually have poor prognosis and a high mobility of long-term disability including PTSD, cognitive deficits, and neurobehavioral deficits [2, 3]. The primary hit of TBI mainly accounts for the severity of the outcomes; however, the secondary hit, such as cerebral edema, oxidative stress, inflammation, excitotoxicity, breakdown of BBB, DNA damage, and iron overload, might contribute to the advanced pathophysiological exacerbation [4–6].

As previously demonstrated, mitochondrial injury or dysfunction can increase the oxidative stress resulting from the production of ROS [7–9], which causes persistent damage to the brain tissue after TBI [10]. Exhaustion of the innate antioxidation and disturbance of the redox system always result in severe consequences; moreover, the high content of polyunsaturated fatty acids in the brain tissue might easily suffer from free radical attacks. The dysfunction can trigger the cascade of cell apoptosis involving Bcl-2 family, cytochrome c, and so on, which induces the peroxidation of lipids and proteins, the oxidation of nucleic acids, and DNA breakdown [11–13]. Hence, the efficacious elimination of ROS and the mitochondrial rebiogenesis after encountering deleterious pathophysiological insults can reverse cellular dysfunction and promote homeostasis [14–16].

The peroxisome proliferator-activated receptor (PPAR) *γ* coactivator-1 (PGC-1) family of transcriptional coactivators is a well-known molecular switch in a variety of metabolic pathways. PGC-1*α* is the family member that facilitates as a transcriptional coactivator that regulates the genes that are involved in energy metabolism through activation of Tfam [17, 18]. It directly links external stress to the regulation of mitochondrial biogenesis and function [19].

SS-31 (D-Arg-Dmt-Lys-Phe-NH2; Dmt-2′,6′-dimethyltyrosine) is a novel mitochondria-targeted peptide [20, 21]. It can penetrate the brain-blood barrier and then locate in the mitochondrial inner membrane independent on mitochondrial membrane potential (MMP) [22]. SS-31 severs as an antioxidant to obliterate the excessive ROS when cellular homeostasis is disturbed. It was mainly researched in the field of ischemic stroke [20], cognitive disorders [23], spinal injury [24], and so on. The role of SS-31 in traumatic brain injury is seldom illustrated, and the potential pathways in mitochondrial function associated with SS-31 are urgent to be uncovered.

Accordingly, it is of interest to explore the neuroprotective role of SS-31 and the mechanisms under it. In this study, we hypothesize that SS-31 can restore the mitochondrial function to alleviate the neurological disorders through the PGC-1*α* pathway after TBI in mice.

## 2. Materials and Methods

### 2.1. Animals

Male ICR mice aged 6~8 weeks and weighted 28~32 g were purchased from the Animal Center of Jinling Hospital, Nanjing, China. All experimental procedures and protocols were reviewed and approved by the Animal Investigation Ethics Committee of Jinling Hospital and were conformed to the Guide for the Care and Use of Laboratory Animals by the National Institutes of Health (NIH). The mice were housed at the hospital's animal facility, which monitors room temperature, humidity, and 12 h light/dark cycle.

### 2.2. Model of TBI

The TBI model in the present study is well recognized and is described by Flierl et al. [25] and our previous study [26, 27]. This Marmarou weight-drop model is done as follows in brief. The mice were anesthetized with chloral hydrate (1%, 5 ml/kg, i.p.). When succeeded, the mice were placed on the weight-drop device. The scalp was incised longitudinally for about 1.5 cm in the middle to expose the skull. After locating the left anterior frontal area as the impact area, a 200 g weight was released onto the skull from the height of 2.5 cm. Then, the surgical incision was sutured with standard procedure. The mice were put back to their cage when all were done. Sham mice sustain the same procedure without the weight drop.

### 2.3. Experimental Protocols

The mice were randomly assigned to one of the following groups: sham, TBI, TBI + vehicle, and TBI + SS-31. Each group had thirty mice: six for brain edema; six for immunohistochemistry, Prussian blue stain, and TUNEL analysis; six for measurement of ROS; six for purifying of mitochondria; and six for Western blot of total protein and hippocampus protein. The mice in the TBI + vehicle and TBI + SS-31 group received 0.9% saline (vehicle) or SS-31 (5 and 10 mg/kg, China Peptides Co., Shanghai, China) 30 min after TBI intraperitoneally. All the mice of each group were euthanized at 24 h post-TBI.

### 2.4. Tissue Processing

The tissue that contained the entire impact brain territory was analyzed for Western blot and biochemistry indexes. The hemisphere tissue was harvested for brain water content analysis. The whole brain tissue need to be immersed in 4% paraformaldehyde overnight for immunohistochemistry, immunofluorescence, Prussian blue, and TUNEL analysis.

### 2.5. Grip Test and Brain Water Content

Grip test was employed to evaluate the gross vestibulomotor function [26]. The test device was made of a thin, horizontal, metal wire that was suspended between two vertical poles 45 cm above a foam pad. The detailed rubrics are listed as follows: (1) mice gripped the wire less than 30 s (0 point), (2) mice held onto the wire without both forepaws and hind paws together (1 point), (3) mice held onto the wire with four paws without tail (2 points), (4) mice gripped the wire with four paws and tail (3 points), (5) mice walked along the wire on all four paws plus tail (4 points), and (6) mice that scored four points also ambulated down from the posts used to support the wire (5 points).

Brain water content was performed by wet/dry ration method [27]. Mouse brain was removed and placed on a precooled operating table 24 h after TBI. The brain stem and cerebellum were taken away, and the hemisphere tissue of the injury site was weighed immediately following removal to acquire wet weight (ww). After drying at an 80°C oven for 72 h, the ipsilateral tissue was weighed to obtain dry weight (dw). The brain water content was calculated as a percentage using the following formula: (ww − dw)/ww∗ 100%.

### 2.6. Isolation of Mitochondria

Mitochondria were extracted following the instructions of the manufacturer with the use of mitochondrial isolation kit for tissue (Beyotime Institute of Biotechnology, Nantong, China). In brief, the contusion area of the brain was ground in ice-cold isolation buffer and then centrifuged at 1000*g* for 5 min. After collecting the supernatants, they were centrifuged at 3500*g* for 12 min at 4°C to precipitate the mitochondria.

### 2.7. Western Blot

With the impact brain tissue lysis, equal amounts of total protein, hippocampus protein or mitochondrial protein were separated by 10% or 12% SDS-PAGE gel and then transferred to PVDF membrane. The target primary antibodies were incubated for 10 h. Subsequently, the corresponding secondary antibodies were incubated for 2 h after TBST washing. The dilution ratio of used antibodies was listed as follows: eIF2*α* (1 : 1000, CST), p-eIF2*α* (1 : 1000, CST), Bcl-2 (1 : 1000, CST), Bax (1 : 1000, CST), cytochrome c (1 : 1000, CST), P53 (1 : 500, Proteintech), PGC-1*α* (1 : 2000, Proteintech), SIRT1 (1 : 1000, CST), H3 (1 : 1000, CST), and *β*-actin (1 : 5000, Bioworld).

### 2.8. Mitochondrial MDA and SOD Content

Determination of mitochondrial malondialdehyde (MDA) and superoxide dismutase (SOD) contents was performed by following the manufacturer's instructions (Nanjing Jiancheng Biochemistry Co., Nanjing, China). Bicinchoninic acid (BCA) method was used to test mitochondrial protein concentration. The content of MDA was calculated as nmol/mg protein, and the activity of SOD was expressed as U/mg protein.

### 2.9. Immunohistochemical Staining and Immunofluorescence

For immunohistochemistry analysis, the peripheral impact tissue sections (4~6 *μ*m) were incubated with anti-PGC-1*α* antibody (1 : 300, Millipore) and anti-SIRT1 antibody (1 : 200, CST) overnight at 4°C. The sections were incubated with horseradish peroxidase-conjugated IgG (1 : 500, Santa Cruz) for 1 h followed by washing in phosphate-buffered saline (PBS) for 2 times, 10 min each. The target proteins were stained with diaminobenzidine (DAB), and cell nuclei were counterstained with hematoxylin.

For immunofluorescence analysis, sections (4~6 *μ*m) were incubated at 4°C with 8-OHdG antibody (1 : 100, CST) and anti-NeuN (1 : 100, Millipore) overnight. The sections were incubated with appropriate secondary antibodies (Alexa Fluor 594, 1 : 200) for 1 hour at room temperature after being washed in PBS for 2 times, 10 min each. Cell nuclei were counterstained with DAPI (1 : 1000, Sigma). All the brain sections were observed in six cortical microscopic fields (400x) by two investigators blind to the grouping.

### 2.10. Reactive Oxygen Species (ROS) Content

Intercellular ROS content was detected with dihydroethidium (DHE) staining according to the manufacturer's instructions (Sigma-Aldrich, St Louis, USA). Briefly, the frozen sections were incubated in DHE which was diluted in PBS at 37°C for 30 minutes under dark conditions, and then the sections were washed in PBS (PH7.4) for 3 times, 5 minutes each. All the immunofluorescence images were collected under the same scan condition. The mean relative fluorescence intensity for each group of tissues was then measured using the Image-Pro Plus system (version 6.0).

For the measurement of mitochondrial ROS, mouse brain tissues weighed at 10 mg were dispersed to single cell suspension by a pipettor. We washed the cells with 10 mM PBS two times and diluted the 5 mM MitoSOX™ (Thermo Fisher, Waltham, USA) reagent stock solution in HBSS/Ca/Mg buffer to make a 5 *μ*M MitoSOX reagent working solution. 1.0 ml of 5 *μ*M MitoSOX reagent working solution was applied to incubate the cells for 10 minutes at 37°C, protected from light. And then the fluorescent intensity of samples was read on the microplate reader (Molecular Device, SpectraMax iD3) at ex/em = 510/580 nm.

### 2.11. Terminal Deoxynucleotidyl Transferase-Mediated dUTP Nick End Labeling (TUNEL) Analysis

The apoptotic cells were observed by fluorescence stain using a TUNEL detection kit (Roche, Indianapolis, USA) according to the method described elsewhere [27]. The extensive DNA fragmentation of the brain sections was featured with condensed nuclei. All the brain sections were observed in six cortical microscopic fields (400x) by two investigators blind to the grouping.

### 2.12. Prussian Blue Staining

The peripheral impact brain sections (4~6 *μ*m) were incubated in the dark with Prussian blue staining solution (2% K_4_[Fe(CN)_6_] and 2% HCl) for 1 h and then incubated with fresh-made diaminobenzidine solution (30 mg DAB, 40 ml 1 M Tris pH 7.5, 1 ml 3% H_2_O_2_) after washing in PBS for 3 times, 5 min each. Finally, the sections were photographed in the magnification of 400x by two investigators blind to the grouping.

### 2.13. Statistical Analysis

SPSS 17.0 (SPSS Inc., Chicago, USA) was used for statistical analysis. Data are expressed as mean ± SEM and evaluated by ANOVA and Tukey's post hoc tests for multiple comparisons. *P* < 0.05 was considered as a gauge of statistical difference.

## 3. Results

### 3.1. SS-31 Alleviated Brain Edema and Restored Mice Sensorimotor Deficit after TBI Insult

**Figure 1:**
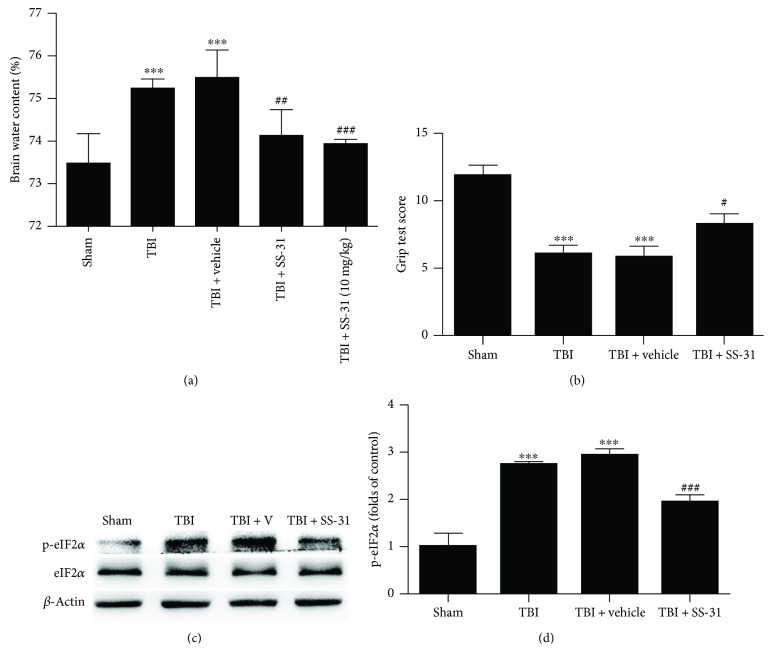
Administration of SS-31 showed neuroprotection after TBI. (a) The mice were grouped into sham, TBI, TBI + vehicle, and TBI + SS-31. The injection doses of SS-31 were 5 mg/kg and 10 mg/kg. The brain samples were collected 24 h post-TBI to evaluate the water content. Both 5 and 10 mg/kg decreased the water content compared with the TBI + vehicle group; however, there was no significant difference between the two doses. *n* = 6 per group. (b) The grip test score in the SS-31 (5 mg/kg as the effective dose) administration group was significantly upregulated than that in the vehicle group. *n* = 6 per group. (c, d) The long-term recovery indicator p-eIF2*α* was restored after treatment with SS-31. Relative protein levels were normalized to the level of *β*-actin and then were measured by ImageJ. Data are presented as mean ± SEM; ^∗∗∗^*P* < 0.001 versus the sham group; ^#^*P* < 0.05, ^##^*P* < 0.01, ^###^*P* < 0.001 versus the TBI + vehicle group.

It is well-known that the brain of the mice may show a significant increase of edema and the sensorimotor function deficit is obvious due to the TBI insult. To evaluate the neuroprotection role of SS-31, we performed the brain water content analysis. Surprisingly, we found that the mice treated with SS-31 half an hour post-TBI impact showed a significant decrease of brain edema (Figure 1(a)), while the brain water content in the TBI + vehicle group showed no difference with the TBI group. As previously reported, SS-31 in a dose of 5 mg/kg acquired enough curative effect [20]. We then used a larger dose of 10 mg/kg to testify its function; however, it did not exhibit a better neuroprotection following TBI (Figure 1(a)).

To assess functional preservation and recovery, the grip test was performed as described above. The grip test was performed in triplicate, and a total score was calculated for each mouse. SS-31 was successful in abrogating the motor functional deficit to some extent (Figure 1(b)). A newly reported biomarker to reflect the long-term cognitive deficit, phosphorylation of the translation initiation factor eIF2*α* (p-eIF2*α*) in the hippocampus [28], was detected by Western blot analysis. As shown, hippocampus p-eIF2*α* was significantly decreased after SS-31 treatment (Figures 1(c) and 1(d)). Therefore, our data demonstrated that SS-31 showed neuroprotection against TBI and 5 mg/kg is the best dose for the following experiments.

### 3.2. SS-31 Preserved the Neuronal Survival

**Figure 2:**
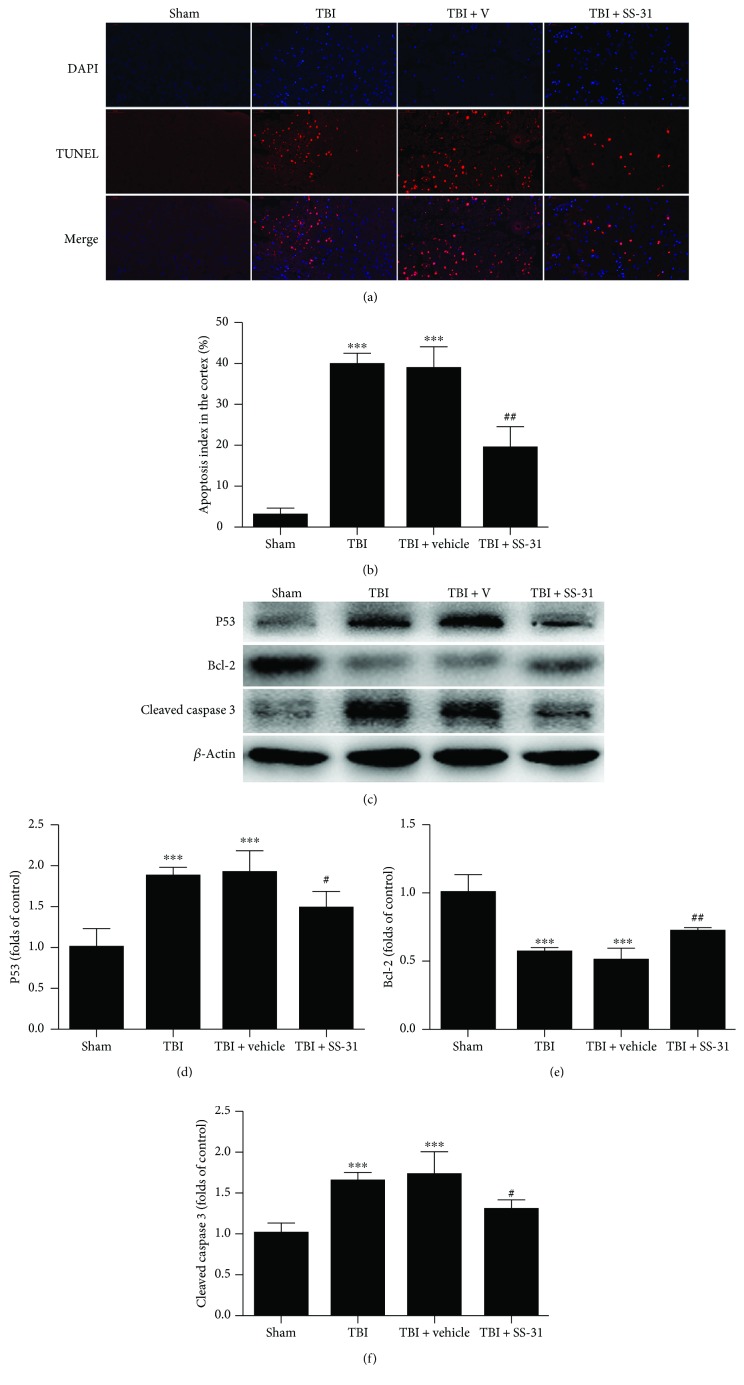
Treatment with SS-31 alleviated the neural apoptosis. (a, b) The TUNEL assay was employed to analyze the apoptotic index 1 day after TBI. SS-31 treatment significantly reduced the percentage of apoptotic neurons. *n* = 6 per group. (c) Western blot showed the pro- and antiapoptotic proteins in the contusion cortex. (d–f) Expression of the P53, Bcl-2, and cleaved caspase 3 protein levels were normalized to the level of *β*-actin. P53 and cleaved caspase 3 were significantly decreased while the Bcl-2 protein level was increased in the TBI + SS-31 group compared with the vehicle group. *n* = 6 per group. Scale bar 50 *μ*m. Data are presented as mean ± SEM; ^∗∗∗^*P* < 0.001 versus the sham group; ^#^*P* < 0.05, ^##^*P* < 0.01 versus the TBI + vehicle group.

The neurons in the impact territory suffered from destructive hit to cause an apoptotic cascade. To evaluate the neuroprotective role of SS-31 in the histopathology level, a TUNEL analysis was employed. The results elaborated that the sham group showed a few TUNEL-positive (stained in red) cells, while the TBI and TBI + vehicle groups exhibited a great amount of apoptotic cell surrounding the cortical contusion up to nearly 40% (Figures 2(a) and 2(b)). However, the apoptosis index in the TBI + SS-31 group fell to about 19.2% (Figures 2(a) and 2(b)). Cleaved caspase 3, P53, and Bcl-2 were detected by Western blot to show the apoptosis in the protein level. The bands were quantified by ImageJ, and there were a significant increase of the antiapoptotic protein Bcl-2 and a significant decrease of the proapoptotic protein P53 and cleaved caspase 3 (Figures 2(c) and 2(d)). These results indicated that SS-31 treatment post-TBI could result in less cell death in the impact territory and harbored the potential to ameliorate the secondary hit following TBI.

### 3.3. SS-31 Reduced Oxidative Stress in the Injured Brain

SS-31 is a novel mitochondria-targeted tetrapeptide that has been shown to scavenge various reactive oxygen species (ROS). Mitochondrial dysfunction could increase the oxidative stress resulting from the production of ROS. Thus, targeting ROS production may be a therapeutic way of TBI.

**Figure 3:**
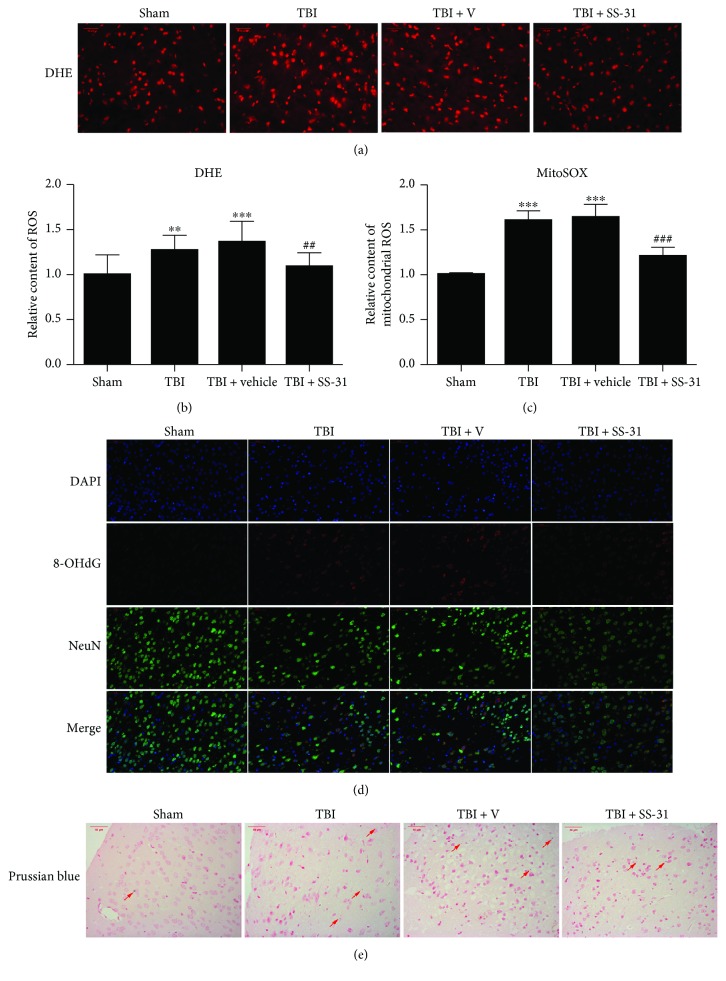
SS-31 scavenged ROS from the root of mitochondria. (a, b) SS-31 administration repressed the production of intercellular ROS detected by DHE staining and subjected to fluorescence microscopy analysis. The mean relative fluorescence intensity was calculated. SS-31 treatment decreased the total ROS content compared with the vehicle group. *n* = 6 per group. (c) The mitochondrial ROS was detected by MitoSOX with the fluorospectrophotometer, and the production of mitochondrial ROS was significantly decreased after SS-31 administration. *n* = 6 per group. (d) 8-OHdG was the indicator of DNA oxidative impairment. SS-31 reduced the formation of 8-OHdG. *n* = 6 per group. (e) Prussian blue staining showed the iron overload cells were fewer in the SS-31 group compared with the vehicle group. Arrows showed the Prussian blue staining. *n* = 6 per group. Scale bar 50 *μ*m. Data are presented as mean ± SEM; ^∗∗^*P* < 0.01, ^∗∗∗^*P* < 0.001 versus the sham group; ^##^*P* < 0.01, ^###^*P* < 0.001 versus the TBI + vehicle group.

The relative level of total intercellular ROS production was visualized by DHE fluorescence, and the contusion area in the TBI and TBI + vehicle groups showed an obviously higher DHE fluorescence intensity compared with that in the sham group (Figures 3(a) and 3(b)). After the administration of SS-31, the total ROS level was significantly decreased (Figures 3(a) and 3(b)). Next, we analyzed the mitochondrial ROS content with MitoSOX staining. The relative content of mitochondrial ROS content was significantly decreased after SS-31 treatment (Figure 3(c)), which was consistent with the result of DHE fluorescence.

8-Hydroxy-2 deoxyguanosine (8-OHdG) is a well-recognized marker of DNA impairment, which is caused by ROS attack [29, 30]. 8-OHdG was detected in or around the nucleus when TBI happened. To testify the 8-OHdG post-TBI, immunofluorescence assay was employed. 8-OHdG (stained in red) was less produced in the TBI + SS-31 group compared with the vehicle group (Figure 3(d)).

Iron overload is frequently reported following TBI [31], which exhibits neurotoxicity due to its ability to form free radicals and induces oxidative stress via the Fenton reactions. In turn, the oxidative stress may aggravate the iron overload. Here, we found that the number of Prussian blue stain-positive cells in the SS-31 administration group was fewer than that in the vehicle treatment group (Figure 3(e)). Taken together, these data suggest that SS-31 protects the neurons from the cytotoxicity of oxidative stress after TBI.

### 3.4. SS-31 Exerted Protective Role in the Mitochondria

**Figure 4:**
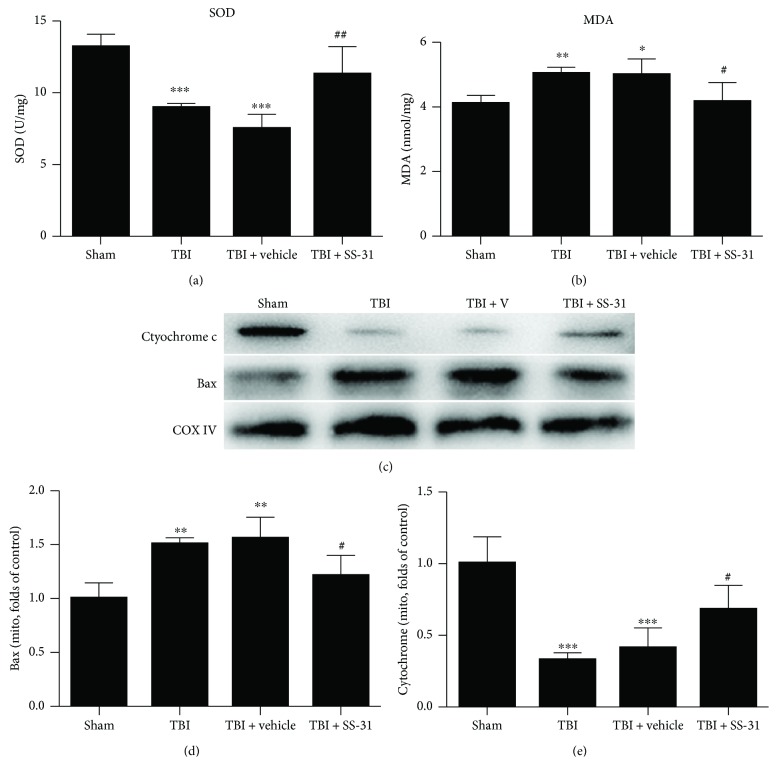
SS-31 reserved the mitochondrial function. (a, b) The mitochondrial SOD activity and MDA content represented the mitochondrial oxidative homeostasis. SOD was increased; inversely, MDA was reduced after SS-31 treatment. *n* = 6 per group. (c) Bax was the mitochondrial proapoptotic protein and cytochrome c was the mitochondrial intrinsic protein. Western blot analysis showed that Bax was decreased while cytochrome c was restored after SS-31 administration. (d, e) Relative protein levels were normalized to the level of COX IV and then were measured by ImageJ. *n* = 6 per group. Data are presented as mean ± SEM; ^∗^*P* < 0.05, ^∗∗^*P* < 0.01, ^∗∗∗^*P* < 0.001 versus the sham group; ^#^*P* < 0.05, ^##^*P* < 0.01 versus the TBI + vehicle group.

Next, to further elucidate the antioxidative role of SS-31 in the mitochondria, we examined two bioindicators that reflect the mitochondrial function. MDA represents the level of lipid peroxidation while SOD catalyzes O_2_^−^ into H_2_O_2_ and then is degraded. Our investigation underlined that the relative level of MDA was significantly elevated in the TBI and TBI + vehicle groups, while the mice in the TBI + SS-31 group restored the production of MDA (Figure 4(a)). In contrast, TBI insult downregulated the activity of SOD and SS-31 administration could regain its bioactivity (Figure 4(b)).

We employed the Western blot analysis to elaborate the improvement of mitochondrial function. Our observation showed that Bax in the mitochondria was elevated in the TBI + vehicle group and went down after the injection of SS-31. Conversely, mitochondrial cytochrome c level was increased in the TBI + SS-31 group compared with the vehicle group (Figures 4(c) and 4(d)).

### 3.5. SS-31 Promoted PGC-1*α* Nuclear Translocation

**Figure 5:**
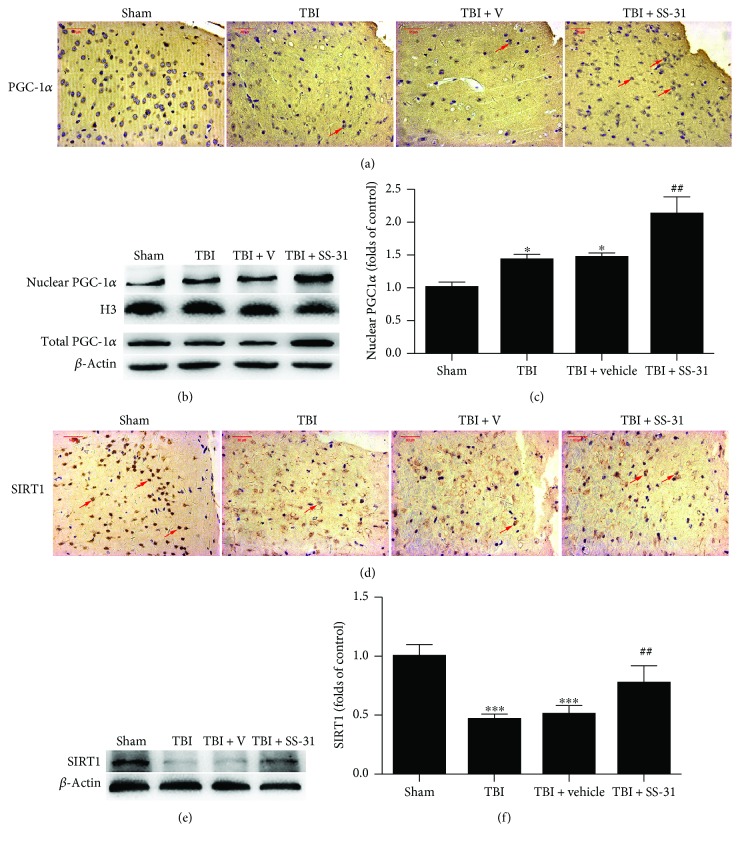
SS-31 promoted the mitochondrial biogenesis potentially via the PGC-1*α* pathway. (a, d) Immunohistochemical staining showed the nuclear PGC-1*α* and SIRT1 positive neurons which were marked by the red arrows. (b) Western blot assay showed the nuclear and total PGC-1*α* expression. (c) Relative protein levels of PGC-1*α* were normalized to the level of H3. These showed the nuclear translocation of PGC-1*α* after SS-31 treatment. (e) Western blot assay showed the restoration of SIRT1 expression. (f) Relative protein levels of SIRT1 were normalized to the level of *β*-actin. *n* = 6 per group. Scale bar 50 *μ*m. Data are presented as mean ± SEM; ^∗^*P* < 0.05, ^∗∗∗^*P* < 0.001 versus the sham group; ^##^*P* < 0.01 versus the TBI + vehicle group.

Finally, for better understanding the potential mechanism of the restoration of mitochondrial function, we analyzed the expression of PGC-1*α*. Our observation showed that PGC-1*α* translocated into the nucleus after TBI and SS-31 administration increased the relative protein level of PGC-1*α* in the nucleus thus enhancing its binding activity to promote mitochondrial biogenesis and function (Figures 5(a)–5(c)). The total PGC-1*α* level was upregulated in the TBI + SS-31 group as well (Figure 5(b)).

SIRT1 is the upstream of PGC-1*α* and regulates the deacetylation of PGC-1*α* to facilitate its transcriptional activity. As shown in Figure 5(d), SIRT1 was reduced following TBI. Except that, compared with the TBI + vehicle group, the TBI + SS-31 group exhibited significantly increased SIRT1 protein level (Figures 5(e) and 5(f)), which indicated that SS-31 enhanced the PGC-1*α* level potentially through the regulation of SIRT1.

## 4. Discussion

Mitochondria are intracellular organelles that function as the “power plant” to generate ATP, known as oxidative phosphorylation (OxPhos) [32]. The study of mitochondria also focuses on cellular homeostasis as well as regulatory mechanisms such as apoptosis and oxidative stress, which have been closely related to the secondary injury of pathophysiologic process post-TBI. Approximately 95% ATP is generated in the mitochondrial cristae as well as the byproduct ROS. Additionally, the neurons are more vulnerable to oxidative damage for the reason that (1) neurons have less endogenous antioxidant than other cell types [33], (2) the brain consumes nearly 20% of total oxygen supply which has been implicated with more susceptibility to oxidative stress, and (3) the oxidative damage will deposit in the postmitotic neurons without regeneration ability. Henceforth, when suffering from TBI, the neurons underwent necrosis and apoptosis with the subsequent mitochondrial dysfunction [34]. As a consequence, the overproduction of ROS and exhaustion of antioxidants enlarged the oxidative stress.

The primary injury of TBI is the mechanical impact-caused contusion whereas the second injury arose from ROS overproduction to a large extent and the subsequent neural death, which causes the majority of TBI-associated brain damage. As mentioned above, mitochondria are the profound organelles where ROS originates; as a fact, the intricate mitochondrial dysfunction requires treatment that specifically addresses the secondary injury [16]. Several recent studies have been focused on the Chinese traditional herbals to research their antioxidative activities and have shown positive outcomes. In this present study, the mitochondria-targeted ROS scavenger SS-31 was applied in the mild TBI mice model. Compared with the flavonoids herbals, SS-31 served as the antioxidant that erased the free radicals from the mitochondrial root. Importantly, SS-31 had the potential to penetrate the blood-brain barrier (BBB) and concentrates >1000-fold in the mitochondrial cristae [21]. The ability of SS-31 in ROS scavenging was confirmed in our research.

Accordingly, we demonstrated multiple key findings after SS-31 administration in the TBI mice model to show its neuroprotective role. Firstly, in the histological and behavioral aspect, SS-31 provided neuroprotection in mice subjected to TBI especially in brain edema and motor competency and predicted cognitive recovery. Secondly, in the pathophysiology aspect, the neural death was relieved by SS-31, which was accounted for the ROS scavenged by SS-31. Finally, in the molecular aspect, SIRT1/PGC-1*α* pathway may contribute to the biogenesis of mitochondria and the restoration of mitochondrial dysfunction after the administration of SS-31. However, there are still a lot of concerns about the research procedures and our observations mentioned above.

We performed the grip test to evaluate the motor function, and the test was developed on the basis of the test of gross vestibulomotor function. In order to better evaluate the neurological impairment after TBI, the neurological severity scores (NSS) are recommended by the canonical TBI protocol [25] and several other studies [26, 27]. We considered the grip test was enough to show its motor function especially in lamb strength, walking, and balance ability.

The improvement of cognitive deficits was analyzed by the p-eIF2*α* protein level in the hippocampus, and it was reported as a promising approach for the treatment of chronic cognitive dysfunction after TBI by Chou and his colleagues [28]. The intrinsic drawback of the weight-drop TBI model was that it only mimicked the acute phase of the TBI secondary injury, but the TBI patients might suffer long-term neurological impairments such as cognitive problems. Besides, the canonical TBI protocol mentioned that when mice were exposed to a weight falling height of 3 cm, they started to spontaneously recover 24 h after trauma, reaching baseline levels only 7 d after TBI [25]. As a result, the cognitive tests such as the water maze and fear conditioning test were not suitable in this TBI model. So we used the p-eIF2*α* protein level as the biomarker to assess the long-term cognitive recovery. In sepsis-associated encephalopathy (SAE), Wu and his colleagues reported that SS-31 reversed the mitochondrial dysfunction and ultimately reversed the cognitive deficits in the SAE mice as well [23], suggesting that SS-31 indeed had the potential to improve the cognitive function.

Intriguingly, we found that iron overload was relieved after SS-31 administration owing to the restoration of mitochondrial dysfunction. The lysis of erythrocytes contained hemoglobin, which gained access to neurons via CD163 [35]. However, neurons had inadequate ferritin level and iron-export protein thus increasing its neurotoxicity [36]. Iron metabolism was mainly conducted in the mitochondria where it formed Fe-S cluster in Complex I and Complex II of ETC. A recent study demonstrated that SS-31 boosted the biogenesis of Fe-S cluster thus facilitating ATP synthesis in the FRDA cell model [37]. A further study needs to explore the energy metabolism after SS-31 treatment in the TBI mice model.

The oxidative stress after TBI resulted in the opening of mitochondrial permeability transition pore (mPTP) that can lead to ROS release to the cytoplasm. In addition, these mPTP channels were insufficient that induced the ROS-induced ROS burst. These reactions led to the destruction of mitochondria. As a consequence, the proapoptotic protein Bax in the outer membrane accumulated post-TBI [11] while cytochrome c was leaked from the mitochondria that bound with apoptotic protease activating factor (Apaf-1) thus cleaving the procaspase 3 [38], suggesting that the mitochondrial apoptotic pathway was activated. Our observation showed that SS-31 significantly decreased the mitochondrial Bax and MDA meanwhile restored the cytochrome c and SOD level, suggesting that SS-31 quenched ROS originated from mitochondria, thus reducing mitochondrial apoptosis and enhancing the antioxidative ability. The mitochondrial biogenesis might be a potential contributor to these findings.

Consistent with previous reports, PGC-1*α* played a vital role in mitochondrial biogenesis following TBI [18]. PGC-1*α* translocated into the nucleus then activated Tfam and NRF1. NRF1 bound to the consensus-binding sites of Tfam gene and then together acted on the promoters within the D-loop region of mtDNA that initiated the replication and transcription of the mitochondrial genome [7, 39]. Our findings indicated that SIRT1 activated PGC-1*α* through deacetylation; however, the downstream transcriptional factors NRF1 and Tfam had not been testified which needed further verification. Moreover, the activation of PGC-1*α* was crucial for the induction of mitochondrial ROS-detoxifying proteins UCP2 and SOD (SOD2) [40], which was confirmed by our findings.

To sum up, the present study demonstrated that SS-31 provided neuroprotection after TBI by scavenging ROS from the mitochondria, thus improving the mitochondrial function and reducing the secondary TBI injury. Moreover, the potential mechanism was the PGC-1*α* pathway. Further study is still needed to elucidate the neuroprotective role of SS-31 in the neuron scratching TBI model in vitro and to elaborate the underlying mechanism of PGC-1*α* nuclear translocation.

## Data Availability

The data used to support the findings of this study are available from the corresponding author upon request.
